# American Medical Association

**Published:** 1884-08

**Authors:** Jno. S. Marshall


### AMERICAN MEDICAL ASSOCIATION.

### SECTION ON ORAL AND DENTAL SURGERY.

### SESSION OF MAY 8, 1884.

REPORTED FOR THE INDEPENDENT PRACTITIONER BY JNO. S. MARSHALL, M. D.

(Continued from page 391.)

The section was called to order by the chairman.

The minutes of the last meeting were read and approved.

The secretary read the following report of the Committee on "Appointment of Dental Surgeons in the Army and Navy."

*Mr. Chairman and Gentlemen:*

Your committee beg leave to report that, in harmony with the suggestion made by them in their report at the meeting in Cleveland, they have endeavored to secure the co-operation of the Surgeons-General of the Army and Navy in gathering statistics in regard to dental diseases prevalent among the soldiers and sailors of the United States. We have the pleasure to report that our request was not only courteously received, but both gentlemen promised to give it careful consideration.

Your committee suggested that the best means of gathering these statistics was to amend the blank reports of the Surgeons by the incorporation of such additional items in the present reports, under the head of Diseases of the Teeth, as would secure the information desired, and at the request of the Surgeons-General a list of items was prepared.

The matter is still under advisement, and we are expecting in due time to receive information of its further progress.

Your committee desire also to express their appreciation of the valuable services rendered them by Dr. Edward Maynard, of Washington, D. C.; all of which is respectfully submitted.

W. W. ALLPORT, *Chairman*,
JACOB L. WILLIAMS,
JOHN S. MARSHALL.

Dr Friedrichs moved the acceptance of the report, and that the committee be continued.

Dr. Williams presented, as chairman of the Committee on Resolutions on the death of Prof. Samuel D. Gross, the following resolutions:

WHEREAS, In the fullness of years and after a life of devoted study and most distinguished services as a practitioner, lecturer, and writer on surgery, it has pleased an all-wise Providence to remove from his earthly labors Prof. Samuel D. Gross, one of the most eminent surgeons that America has ever produced; and—

WHEREAS, He was largely instrumental in securing the establishment of the section on Dental and Oral Surgery in the American Medical Association; and—

WHEREAS, By his years of uniform interest and efforts in behalf of a thorough education of dental surgeons in general medicine, he has placed our department of practice under enduring obligations to him; therefore be it

*Resolved*, That the medical profession has lost one of its most eminent and useful members, and our section mourns the loss of one of its warmest and most devoted friends.

*Resolved*, That the foregoing preambles and resolutions be inscribed upon the minutes of the Association, and that the secretary be instructed to transmit a copy to the family of the deceased.

JACOB L. WILLIAMS, *Chairman*,
W. W. ALLPORT,
GEO. J. FRIEDRICHS,
TRUMAN W. BROPHY.

On motion the resolutions were unanimously adopted.

*Dr. Marshall*—Stated that it had been the custom of the section to recommend to the Nominating Committee of the Association the names of those gentlemen whom it desired for its officers, and as the committee would make its final report as *per programme* on Friday morning, it would be well if the section would make such recommendations at this meeting. He therefore moved that the section proceed to take an expression of the wishes of the members by an *informal* ballot for chairman and secretary. The ballot resulted in recommending Dr. W. W. Allport, of Chicago, Ill., for chairman, and Dr. Edward C. Briggs, of Boston, Mass., for secretary.

*Dr. Friedrichs*—Said that the recommendations would come too late, for the Nominating Committee had made its final report this morning.

*Dr. Williams*—Could not see why the committee should have reported the names of section officers without a conference with the section; it was entirely unprecedented, and he could not understand it.

*Dr. Harlan*—Said *he* had been chosen chairman, and Dr Mears, of Philadelphia, secretary of the section, by the Nominating Committee, and thought the action of the section was very unwise, but in view of the preference manifested, would decline in favor of Dr. Allport.

*Dr. Marshall*—Asked if Dr. Mears had been present at any of the sessions of the Association.

(*Dr. Stellwagen*—No!) According to the by-laws of the Association then the office or secretary is also vacant, and he moved that the Nominating Committee be asked to reconsider its action in nominating officers for this section.

*Dr. Williams*—Moved that the secretary be instructed to at once report the action of the section to the chairman of the Nominating Committee, and call his attention to the fact of the absence of Dr. Mears, and ask for a reconsideration of the nominations for this section.

*Dr. Harlan*—The secretary will please also state to Dr. Hooker, the chairman of the committee, that I decline the nomination of chairman.

There being no other miscellaneous business the chairman called for the reading of the paper by Dr. Harlan, of Chicago, Ill., on "The removal of stains from the teeth caused by the administration of medicinal agents and the bleaching of pulpless teeth."

SYNOPSIS.

THE REMOVAL OF STAINS FROM THE TEETH CAUSED BY THE ADMINISTRATION OF MEDICINAL AGENTS AND THE BLEACHING OF PULPLESS TEETH.

BY A. W. HARLAN, M. D., CHICAGO, ILL.

The paper opened with the statement that a large number of medicinal agents administered by physicians temporarily stain the teeth, but very few have any permanent effect. The mineral acids, nitric, sulphuric, hydrochloric, etc., if used for long periods, may temporarily stain the teeth and also have a deleterious effect upon their mineral constituents, but the staining is so slight that no particular methods need be devised to restore their normal appearance. The vegetable acids may likewise be dismissed. The tannates and astringents generally do not permanently stain them, and the same may be said of the ferrum preparations—tobacco, rhubarb, infusion of saffron, carmine, catechue, iodine, and kindred agents. These temporary stains are usually removed with ease by the use of dentifrices, but occasionally it may be necessary to polish them with wooden or leather points charged with fine emery or pumice-stone, followed by powdered rotten stone or precipitated chalk, incorporated with thick solution of white castile soap. In cases where the enamel has been fractured, or the masticating surfaces have been deprived of their enamel by wear, tobacco stains permanently, and there is no remedy for the stain, except to cap the teeth with gold, and this is seldom found necessary unless the ends of the teeth become so sensitive from wear as to need protection.

The single medicinal agent which I have found to stain the teeth permanently is nitrate of silver. Solutions are frequently used in the mouth and throat of too great strength. Many times the powdered nitrate is used to dust diseased mucous membranes, and the teeth often suffer in consequence.

Occasionally dental surgeons use the solid stick in treating the necks of sensitive teeth and to arrest incipient caries. Carelessly used, it causes unsightly stains, and these are the cases we are called upon to treat. No amount of polishing will remove these stains without injury to the external surfaces.

The agents which may be used are not numerous. Cyanide of potassium, iodide of potassium, tinct. of iodide, and liquor ammonia fortis are all recommended. Other substances are used to remove fresh stains, notably solution of chloride of sodium if used immediately.

I recommend as the neatest, safest, and most certain method to adjust the rubber-dam, dry the teeth and paint them with the compound tincture of iodine, allowing it to dry, then moisten the surfaces with stronger liquor ammonia for two or three minutes, and then wash with peroxide of hydrogen. The stains will disappear in consequence of the chemical change resulting in the formation of iodide of silver and nitrate of ammonia. They are afterwards to be polished in the usual manner.

BLEACHING PULPLESS TEETH.

Most of the methods in general use are faulty, and few are useful in all cases. In order to bleach a pulpless tooth the operator must first fill the root for one-third its length. All decay and fragments of pulp should be removed, but discolored dentine, if hard, need not be cut away. Adjust the rubber-dam over the tooth to be operated upon, including those immediately contiguous; the cavity is first repeatedly washed with $H_2\ O_2$, then carefully dried by the use of the hot blast syringe. A small quantity of chloride of alumina is next placed within the cavity and moistened with peroxide of hydrogen and allowed to remain about five minutes, and then washed out with a clear solution of sodæ biboras, $N\ O_2\ B_4\ O_7 - 10\ H_2\ O$— and thoroughly dried. The tooth in most cases will be found to have resumed its normal color. The change of color has been brought about by the complete oxidation and destruction of the contents of the tubules. A tooth to be bleached should not be soaked with creosote, carbolic acid, alcohol, or any other substance capable of coagulating albumen. When such agents have been used, first wash the cavity with a clear solution of sodæ biboras, and then follow the method already mentioned.

The bleaching of the tooth is brought about by the rapid liberation of chlorine from $Al_2\ Cl_6$, in the presence of $H_2\ O_2$, resulting in the formation of H Cl, and $H_2$ O, leaving unsatisfied O H, and Cl.

In order to maintain the color oxy-chloride of zinc should be used to fill the remainder of the pulp canal, and as much of the cavity of decay as the judgment of the operator will suggest. The oxy-chloride should not be allowed to become moistened with the saliva or other fluid, and as soon as it is sufficiently hard, the balance of the cavity filled with gold. Moisture coming in contact with the oxy-chloride jeopardizes the permanency of the color on account of its speedy absorption of fluids.

DISCUSSIONS ON DR. HARLAN'S PAPER.

*Dr. Friedrichs*—I used to attempt to bleach teeth years ago, but have not made such an effort in the last ten years. If our services are engaged in season, there should be no occasion for bleaching, and I very seldom see a case now that requires such service.

*Dr. Harlan*—I have tried all the known remedies for discolored teeth, and think from such experience that peroxide of hydrogen combined with chloride of aluminum are the most successful agents. It is important, however, that the drugs be good. I think Tromdroff's peroxide of hydrogen and Merk's chloride of aluminum are the most reliable.

*Dr. Friedrichs*—In reference to the stains of nitrate of silver (Ag. N $O_3$), I prefer to remove them by mechanical means rather than make the attempt by the use of chemicals. A stick and pulverized pumice-stone are all that is needed to effectually remove such stains.

*Dr. Brophy*—I would ask Dr. Friedrichs if he does not find in some cases that the stains have penetrated too deeply to be removed by such means.

*Dr. Friedrichs*—I have used this means to a very great extent, and am entirely satisfied with it. I have had a great many patients suffering from "erosion" and find that Ag. N $O_3$ stains do not penetrate beyond the control of such mechanical means as I have mentioned.

(TO BE CONTINUED.)

